# Influences of Spices on the Flavor of Meat Analogs and Their Potential Pathways

**DOI:** 10.3390/foods12081650

**Published:** 2023-04-15

**Authors:** Jingyao Yuan, Fang Qin, Zhiyong He, Maomao Zeng, Zhaojun Wang, Jie Chen

**Affiliations:** 1 State Key Laboratory of Food Science and Technology, Jiangnan University, Wuxi 214122, Chinazhaojun.wang@jiangnan.edu.cn (Z.W.); 2 International Joint Laboratory on Food Safety, Jiangnan University, Wuxi 214122, China

**Keywords:** market survey, extrusion, spices, correlation analysis, antioxidative abilities

## Abstract

This study evaluated the correlation between the sensory characteristics and spices of 50 commercial meat analogs and selected 4 spices to improve the flavor characteristics of soy protein concentrate (SPC) extrudates. Volatile compounds in extrudates and commercial meat analogs were investigated using headspace solid-phase microextraction and gas chromatography–mass spectrometry. The total concentrations of off-flavor volatile compounds in commercial products decreased with an increasing degree of processing. Furthermore, after adding spices during extrusion, the concentrations of volatile compounds such as aldehydes, alcohols, and furans related to thermal treatment decreased by approximately 5–39%, 5–15%, and 11–56%, respectively. Among them, compounds such as nonanal, 2-pentlyufuran, and 1-octen-3-ol, typical off-flavors in soy-based food, decreased by 8–42%, 11–55%, 2–52%, respectively. The correlation analysis between the antioxidative abilities of spices and volatile compounds showed that the contents of total phenolics were negatively correlated with the contents of ketones and alcohols in extrudates (*p* < 0.001). Moreover, the aroma-active compounds in extrudates were changed. More pleasant compounds, including alkanes and olefins, were observed by adding different spices as well. Especially in black pepper treated extrudates, the OAV value of off-flavor volatile compounds such as hexanal, octanal, 2-pentlyfuran decreased. In conclusion, the addition of spices can reduce off-flavor compounds related to thermal reactions, such as oxidation and the Maillard reaction, and impart newly pleasant flavors to extrudates during the extrusion of SPC. It is important to explore new methods that can be used to improve the flavor of extrudates so that consumers’ preferences of meat analog products can be improved.

## 1. Introduction

High-moisture extrusion is frequently used to obtain fibrous extrudate, which is a promising candidate for producing meat analogs. Soy protein concentrate (SPC) has been widely used as a raw ingredient of extrudate owing to its low-cost, high-protein content and quality, good amino composition, and comparable digestibility [1,2]. However, the off-flavor of plant-based meat analogs decreases consumers’ acceptance [3].

Off-flavor problems in soy proteins can be improved using some strategies, such as cultivating soybeans with lower lipoxygenase activity [4,5], fermentation (molds and bacteria) to produce extra compounds with pleasant flavor [6,7], and embedding off-flavor compounds with hydrophobic substances [8,9]. Some of them have been used in practical production, and the off-flavor compounds in soy protein can be reduced to some extent. However, under severe process conditions, that is, elevated temperature processes, the protein structure unfolds to allow the release of more off-flavor compounds [10,11,12,13]. Additionally, some other reactions including oxidation and the Maillard reaction may generate more off-flavor compounds [14,15]. However, research on the direct flavor improvement methods of extrudates made from soy protein was limited.

Previous study reported that the polyphenol in avocado peel extract could inhibit oxidation in the production processes of soy-based burgers [16]. It has been reported that several spices have plenty of polyphenols with antioxidative abilities and could help fortify the sensory quality of foods [17,18,19,20]. Black pepper contains bioactive compounds (e.g., phenolics), which can reduce oxidative products in beef [21,22]. Furthermore, black pepper could improve antioxidant properties and preference scores. More than 20 phenolic compounds were noted in red pepper [23,24]. Spices could become the main flavor attribute of food products. It has been reported that adding 0.5% of garlic or onion can improve the sensory characteristics of beef and inhibit lipid oxidation [25]. Dwivedi–Vasavada [26] reported that pepper-treated beef had lower thiobarbituric acid values and reduced rancid odor and flavor. Therefore, we proposed that adding spices during extrusion can reduce off-flavor compound generation and obtain extra pleasant flavor to enrich the flavor profiles of extrudates.

A market survey was performed to explore the probable routes of the generation of off-flavor compounds in meat analog products and potential methods that can be used to improve their flavor profiles. This study aimed to evaluate the correlation between sensory characteristics and spices by analyzing the volatile compounds in 50 commercial meat analog products. Based on the correlation, we selected four spices including black pepper, red pepper, onion, and garlic which have certain amounts of antioxidative abilities, and investigated their effects on the off-flavor compounds and other volatile compounds upon extrusion. The antioxidative abilities of different spices and their influences on the flavor profile of extrudates were investigated.

## 2. Materials and Methods

### 2.1. Materials

SPC (SST-04) was purchased from Yihai Kerry (Shanghai, China). Spice powders (garlic, onion, black pepper, and red pepper) were purchased from McCormick (Shanghai, China). Heat-resistant plastic bags were purchased from Xizhilong (Shijiazhuang, China).

Analytic grade 2-methyl-3-heptanone was purchased from Sigma–Aldrich Co. (St. Louis, MO, USA). Folin–Ciocalteu reagent, 2,2-azinobis-(3-ethylbenzthiazoline-6-sulfonate) (ABTS), 6-hydroxy-2,5,7,8-tetra-methylchroman-2-carboxylic acid (Trolox), and 2,4,6-tris-(2-pyridyl)-s-triazine (TPTZ) were supplied by Sigma–Aldrich Chemical Co. (St. Louis, MO, USA). Methanol (TEDIA, Co., Ltd., Fairfield, OH, USA) was high performance liquid chromatography (HPLC) graded, and the other reagents were analytically graded and purchased from Sinopharm Chemical Reagent Co., Ltd. (Shanghai, China). Ultrapure water was produced by a lab-scale water purification system (Smart-S2-30DH, EPED, Co., Ltd., Nanjing, China).

### 2.2. Plant-Based Meat Analog Products

The 50 plant-based meat analog products were directly purchased from Chinese supermarkets and online shops and classified into textured soy protein (n = 5), meat minces (n = 14), nuggets (n = 9), meat patties (n = 9), and instant vegan snacks (n = 13) according to the seasoning styles of products. Meat analog products were defrosted at room temperature and crushed with a blender before being analyzed.

### 2.3. Extraction of the Phenolic Fract Ion

**Figure 1:**
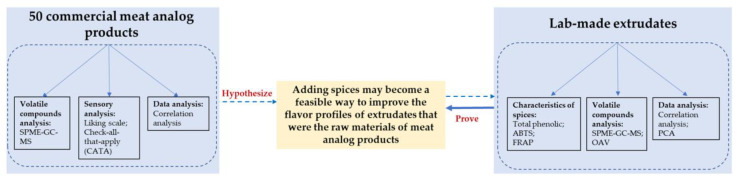
Technical roadmap.

Spices were mixed with 20 mL ethanol for 1 h at 37 °C using ultrasound-assisted technology. Supernatants were collected after being centrifuged at 10,000 rpm for 20 min at 4 °C, and residue extraction was repeated twice as previously mentioned. The supernatants were filtered twice to remove the residues and subsequently used for further analysis. The technical roadmap is shown in Figure 1.

### 2.4. Determination of Total Phenolic Contents

The total phenolic content was quantified using the Folin–Ciocalteu method with some adaptation [27]. Next, 0.25 mL of the sample and 1-mL Folin–Ciocalteu (0.2 M) were mixed and incubated at room temperature for 5 min. Subsequently, 3 mL of 75-g/L Na_2_CO_3_ solution was added and fixed to the volume of 10 mL with distilled water. Absorbance was measured at 765 nm using a microplate reader (Spectra Max 190, Molecular Devices, Sunnyvale, CA, USA) after being incubated for 2 h at room temperature away from light. The total phenolic content was expressed as milligrams of gallic acid equivalent per gram of spices (mg GAE/g dw).

### 2.5. Determination of the Antioxidant Activity of Phenolics Fractions in Spices

The analysis of ABTS radical scavenging activity was estimated using methods described by Shao, Tang [28], and Quan, Qie [29] with minor modifications, and the ABTS radical scavenging activity of samples was expressed as the Trolox equivalent antioxidant capacity in micromoles Trolox per gram dry weight of spices (μM TE/g dw). Here, 7 mmol/L ABTS solution and 2.45 mmol/L potassium persulfate were mixed and incubated at room temperature for 18 h. This working solution was diluted with 0.2-mol/L phosphoric acid buffer at a pH of 7.4 to reach an absorbance of 0.70 ± 0.02 at 734 nm. Ten microliters of the samples were mixed with a 190 µL working solution. The absorbance was measured at 734 nm by using a microplate reader.

The estimation of ferric-ion-reducing antioxidant power (FRAP) was performed as described by Qie, Chen [30] with several modifications. The FRAP working solution was obtained by mixing 10-mmol/L TPTZ in 40-mM hydrochloric acid, 20-mM FeCl_3_ solution, and acetate buffer (0.3 mmol/L, pH = 3.6) at a ratio of 1:1:10 (*v*/*v*/*v*), and the mixture was incubated for 1 h at 37 °C before use. Next, 190 µL FRAP working solution was added to a 10 µL sample for a 30 min incubation, and the absorbance was measured at 593 nm. Trolox solutions were used to construct calibration curves, and total antioxidant capabilities were expressed as µM TE/g dry weight of spices.

### 2.6. High-Moisture Extrusion

SPC and different spices were mixed using a blender at a ratio of 99.5%:0.5% (w:w) before extrusion. The mixture was extruded using a twin screw extruder (ZE, ATS, Co., Ltd., Suzhou, China). There were seven heating zones on the extruder to set the temperature, and the temperature of each zone used was 25 °C, 30 °C, 70 °C, 100 °C, 130 °C, 140 °C, and 130 °C. The screw speed was 110 rpm, the feeding speeds of SPC and water were 0.3 kg/h, and the final water content of extrudates was 50%. A cooling mold was controlled by a water pump (AC 150, Thermo Fisher Scientific, Waltham, MA, USA) at 55 °C, and it was connected at the exit of the extruder to cool down and further shape the extrudates. The extrudates were cooled to room temperature and subsequently sealed in heat-resistant plastic bags.

### 2.7. Solid-Phase Microextraction (SPME) Extraction

Exactly 2.0 g of the samples were put into a 20 mL headspace (HS) vial after being crushed using a mixer. Twenty microliters of 2-methyl-3-heptanone were added as an internal standard. A fiber coated with a 50/30-µm DVB/CAR/PDMS layer (Supelco, Bellefonte, PA, USA) was used to extract volatile compounds in extrudates in a 70 °C water bath for 30 min until they reach equilibrium. Subsequently, the fiber was injected into a gas chromatograph and mass spectrometer and analyzed.

### 2.8. GC-MS Condition

A DB-WAX UI capillary column (30 m × 0.25 mm × 560.25 µm) was used as a GC column. Helium at 1 mL/min was used as a carrier gas. The initial temperature of the oven was maintained at 40 °C for 3 min and subsequently increased to 230 °C at a rate of 5 °C/min.

The mass spectra were operated in electronic impact mode: 70 eV. The ion and transmission line temperatures were 210 °C and 250 °C, respectively. The mass resolution was 25,000, and the weight range was 33–450 amu.

### 2.9. Quantitative Analysis of Volatile Compounds

#### 2.9.1. Calculation of Relative Contents of Volatile Compounds

The relative contents of volatile compounds in pure spices and commercial meat analogs were calculated using the following equation:

(1) $$\mathrm{Relative}\;\mathrm{content}\;(\%)=\frac{\;\mathrm{Chromatographic}\;\mathrm{peak}\;\mathrm{area}\;\mathrm{of}\;\mathrm{compound}}{\mathrm{Total}\;\mathrm{chromatographic}\;\mathrm{peak}\;\mathrm{area}\;\mathrm{of}\;\mathrm{compounds}}\;\times \;100\%$$

#### 2.9.2. Semi-Quantification of Compounds

Additionally, 2-methyl-3-heptanone (10 µg/kg, the solvent was methanol) was used as the internal standard to perform the semi-quantitative analysis of volatile compounds in extrudates added with spices using the following formula:

(2) $$\mathrm{Concentration}\;\mathrm{of}\;\mathrm{compound}/(\mu g/\mathrm{kg})=\frac{A_{1}{\times C}_{1}{\times V}_{1}}{A_{2}{\times W}_{1}}\;\;\times \;100\%$$

where A1 is the chromatographic peak area of the compound, C1 is the concentration of the internal standard (µg/kg), V1 is the volume of the added internal standard (µL), A2 is the chromatographic peak area of the internal standard, and W1 is the sample quality (g).

### 2.10. Aroma-Active Compounds in Extrudates

The odor activity value (OAV) represents the contribution of a specific volatile compound to a flavor. Although the concentrations of some compounds are relatively low in samples, they can still influence the total flavor owing to their low thresholds. OAV was calculated by the concentration of the compound and its aroma threshold (µg/kg). Volatile compounds with a ratio value of ≥1 were considered aroma-active compounds, and the contribution of a specific compound to the flavor profile was proportional to its OAV. The following was the calculation formula of OAV:

(3) $$\mathrm{OAV}=\frac{C}{T}\;\;\times \;100\%$$

where C (µg/kg) is the concentration of the compound and T is the odor threshold value of the compound.

### 2.11. Consumer Sensory Analysis

#### 2.11.1. Liking Scale

Additionally, 42 untrained-panelists were recruited from students in the laboratory. A nine-point structured hedonic scale (9 = liked extremely, 5 = neither liked nor disliked, and 1 = disliked extremely) was used to evaluate panelists’ opinions on the odor of extrudates.

#### 2.11.2. Check-All-That-Apply (CATA)

Check-all-that-apply was utilized as the method to evaluate the sensory characteristics of commercial meat analog products. During the sensory evaluation, another 15 students in addition to the abovementioned 40 students were recruited to provide descriptors applicable to the extrudates. The four most frequently occurring words were warm-over, beany, grassy, and mushroom; they were defined as descriptors for evaluating undesirable flavors of commercial plant-based meat analog products. They were provided to the sensory evaluators for selection in the CATA sensory evaluation. The frequency or number of occurrences of descriptive words were used to evaluate the flavor profiles of commercial meat analog products.

### 2.12. Statistical Analysis

A statistical analysis of data was performed by Analysis of Variance (Statistical Package for the Social Sciences 21.0, SPSS Inc., Armonk, IL, USA). All analyses were performed for three replicates, and data were expressed as means ± standard deviations. Correlation analysis was performed using Origin 2021b software (OriginLab Corporation, Northampton, MA, USA). Principal component analysis (PCA) was performed using the SIMCA software (SIMCA-P + 13.0, UMETRICS, Umea, Sweden), and the first two principal components (PCs) were utilized for PCA calculation. Compounds not detected were input as “0” during PCA.

## 3. Results and Discussion

### 3.1. Analysis of 50 Commercial Plant-Based Meat Analogs

#### 3.1.1. Off-Flavor Compounds in Meat Analogs

Volatile compounds of commercial meat analogs were analyzed by GC-MS, and the relative concentration of compounds were calculated as the ratio of the peak area between the specific compound to the total peak area of all compounds. Twenty-three volatile compounds, as shown in Table 1, with typical off-flavor characteristics related with beany or off-flavor, were identified in commercial plant-based meat analog products (Table 1) including aldehydes (n = 10), alcohols (n = 5), ketones (n = 4), acids (n = 3), and one furan. These compounds were reported as representative compounds that lead to beany or off-flavor in soy-based foods [31,32,33]. The total relative contents of off-flavor volatile compounds in commercial meat analogs were calculated by being added up. 

Hexanal, 1-octen-3-ol, (E, E)-2,4-Decadienal, and (E)-2-octenal were reported to be the main beany flavor compounds in soy milk [34]. Hexanal, 2-pentlyfuran, 1-nonanol, nonanal, and octanal have been reported to be the main volatile beany-flavor compounds in textured soy proteins [35,36,37].

**Figure 2:**
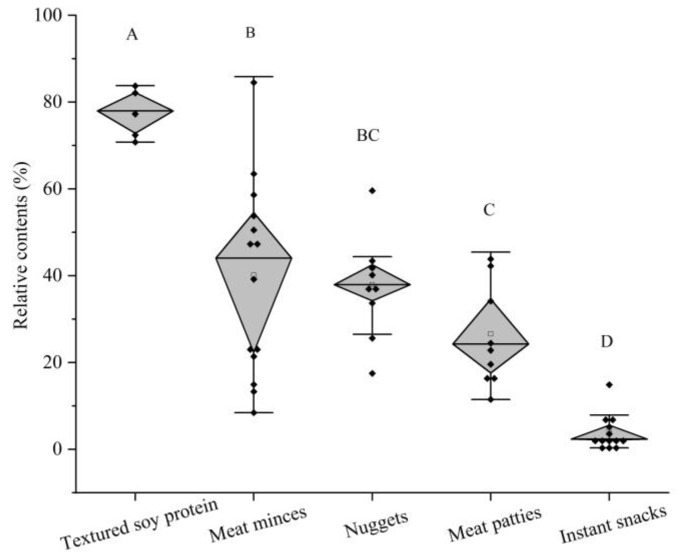
The total relative contents of off-flavors in raw commercial plant-based analog products. Different capital letters indicate significant differences (α = 0.05 level).

The 50 plant-based meat analog products were then divided into 5 categories according to their processing styles and extent of processing. The categories were conducted according to the processing degree of meat analogs, and they were divided into raw-textured soy protein, meat minces, nuggets, meat patties, and instant snacks. The box diagram of the total off-flavor of each sample is shown in Figure 2.

The total relative content of off-flavor compounds significantly varied between different categories. The total off-flavor compounds in raw-textured soy protein were the greatest, ranging from 71% to 84%. The total relative percentages of off-flavor compounds in the other four categories were approximately 8–86%, 18–60%, 11–45%, and 0.3–15%, respectively. In general, owing to the differences between spices and essences used, the total relative amounts of off-flavor compounds varied between samples belonging to the same category.

As shown in Table 1, the remaining off-flavor volatile compounds such as hexanal and 1-octen-3-ol could be detected in commercial meat analog products. Consumers can perceive some of them such as hexanal, nonanal, and 1-hexanol at low contents, owing to their relatively low thresholds. Therefore, the acceptance of these products by consumers would be affected. Products such as chicken nuggets, meat patties, and meat balls were made from textured soy protein by adding essences or spices to mask off-flavor or to mimic the meaty flavor of real meats. However, this method was ineffective because the already existing = off-flavor volatile compounds in textured soy protein, as well as the combination of off-flavor volatile compounds with other food ingredients, could not be easily masked or released. Actions such as inhibiting the generation of off-flavor volatile compounds and replacing the combination of off-flavor volatile compounds and protein with other pleasant volatile compounds could be taken. Both the generation of off-flavor volatile compounds would be retarded, and the retention of off-flavor volatile compounds in meat analogs would be decreased so that flavor profiles of meat analogs could be improved.

#### 3.1.2. Correlation Analysis

**Figure 3:**
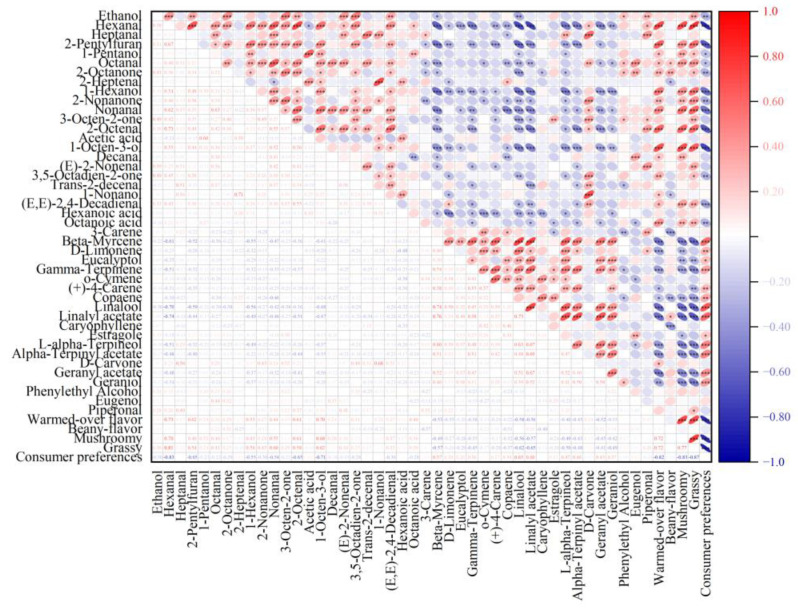
Correlation analysis of commercial plant-based analogs. (* =0.05, ** =0.01 and *** =0.001 level).

Spearman’s correlation analysis was performed to explore the influence of volatile compounds in meat analogs and spices on the sensory profiles. The relationship between different volatile compounds in extrudates and spices and sensory evaluation is presented in Figure 3. Warm-overed, beany, mushroom, and grassy flavors were unexpected flavor profiles in meat analogs. 

The warm-over flavor was a product of lipid oxidation, which could damage consumers’ food acceptance [38]. This flavor was highly positively correlated with the relative contents of off-flavor such as hexanal, 2-pentlyfuran, 1-hexanol, nonanal, 2-octenal, 1-octen-3-ol, and hexanoic acids in meat analog products, and it was highly negatively correlated with substances in spices (e.g., beta-myrcene, D-limonene, gamma-Terpinene, linalool, linalyl acetate, L-alpha-Terpineol, alpha-terpinyl acetate, and geranyl acetate).

The beany flavor was a combination of substances such as hexanal, 2-pentlyfuran, and nonanal, and it was highly negatively correlated with 3-carene and caryophyllene, which was representative compounds in black pepper [39]. Additionally, 1-octen-3-ol and hexanal were the representative volatile compounds of mushroom and grassy flavor, respectively, and their negative influences on flavor would be exacerbated by the synergetic effects of other unpleasant compounds including heptanal, 2-pentlyfuran, octanal, 1-hexanol, nonanal, acetic acid, 1-octen-3-ol, and hexanoic acid. The extent of mushroom and grassy flavors was highly positively correlated with the contents of most of those abovementioned compounds including 2-pentlyfuran, octanal, 1-hexanol, nonanal and 1-octen-3-ol. They were negatively correlated with 3-carene, gamma-terpinene, linalool, linalyl acetate, alpha-terpinene, terpinly acetate, geranyl acetate, and geraniol, and three of them are alkenes. Alkenes have been reported to be the main compounds in spices, such as ginger and black pepper [40].

Moreover, this study conducted a correlation analysis between the relative contents of volatile compounds and consumers’ preference for commercial meat analogs. Consumers’ preferences were negatively correlated with several off-flavor compounds in meat analogs, and they were positively correlated with volatile compounds in spices.

In general, it could be speculated that the influences of spices are not negligible because spices have enhanced the sensory profiles of meat analogs by bringing new pleasant volatile compounds. Furthermore, lipid and free fatty acid oxidation that leads to off-flavor generation was retarded, owing to spices’ antioxidative capacities. Adding spices during processes such as extrusion may become a feasible and efficient method that can not only improve the flavor profiles of meat analogs, but also inhibit flavor deterioration.

### 3.2. Determination of Antioxidant Abilities and Total Phenolics of Spices

Spices were reported to be widely used in seasonings such as flavor-enhancer and antioxidants. Off-flavors compounds including hexanal, (E)-2-octenal, 2-pentlyfuran, and 1-octen-3-ol mainly come from reactions that include lipid and free fatty acid oxidation during processes, such as bean crushing, protein extraction, and high-temperature extrusion, and their generation may be inhibited by antioxidation [41,42]. Antioxidant capacity was one of the main functional properties displayed in phenolic compounds. In the present study, the total phenolics were evaluated using the Folin–Ciocalteu method, and the potential antioxidative capabilities of spices were represented using the ABTS free radical scavenging ability and FRAP to understand the potential capability of a specific spice to retard further flavor deterioration in extrudate production by partially inhibiting oxidation-related reactions.

**Figure 4:**
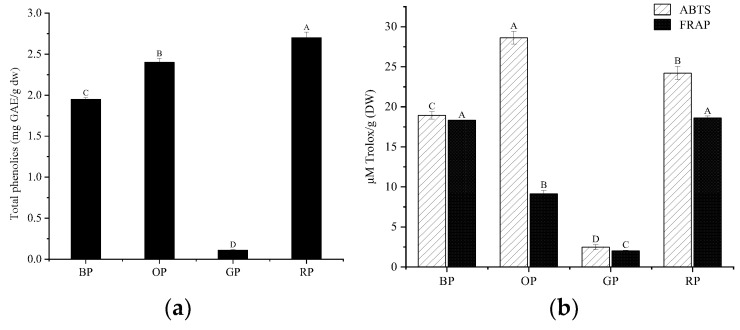
(**a**) Total phenolics in spices; (**b**) antioxidative abilities of spices. Columns with different letters (A–D) indicate significant difference between samples (α = 0.05 level). OP: Onion powder; RP: Red pepper powder; BP: Black pepper; GP: Garlic powder; 2 N.D. means not detected.

As shown in Figure 4a, the total phenolic contents of spices range approximately from 0.11 to 2.7 mg GAE/g (DW). The ABTS values of black pepper, onion, garlic, and red pepper powder were 18.94, 28.63, 2.47, and 24.22 µM Trolox/g (DW). The FRAP values were 18.33, 9.15, 9.15, and 18.61 µM Trolox/g (DW) (Figure 4b). Spices with such antioxidative abilities have the potential to be used as antioxidants in the extrusion to inhibit partial oxidation, and it is one of the main causes of off-flavor volatile compounds such as aldehydes and some alcohols in extrudates. It has been reported that spices retard oxidation and improve the total flavor of several foods. Onions and garlic contain a large amount of an antioxidative phenolic compound called quercetin, which can retard the lipid oxidation of ground beef during cooking [43,44]. Thirty-seven phenolics were noted in black pepper, and the addition of pepper in turkey inhibited the formation of lipid oxidation products by inhibiting 66% of lipid hydroperoxide formation and reducing thiobarbituric acid reactants TBARS values [45].

### 3.3. Flavor Analysis

#### 3.3.1. Volatile Compounds of Spices and Extrudates

**Figure 5:**
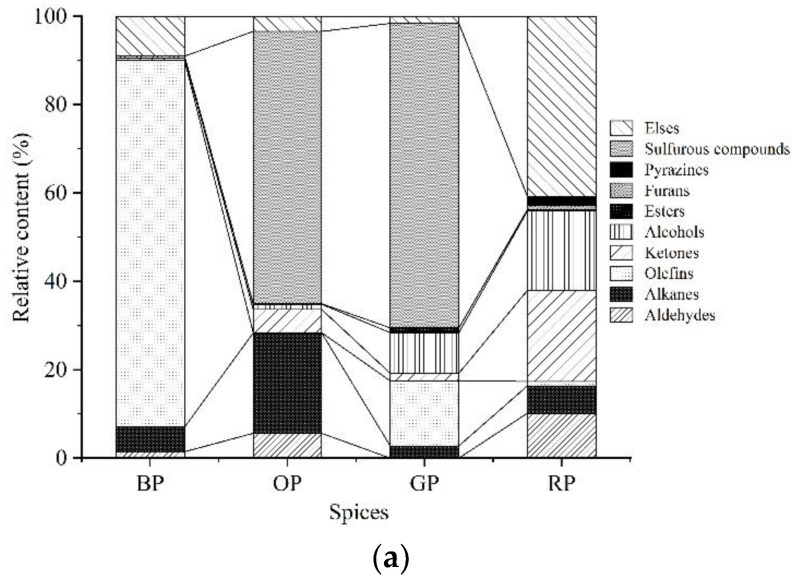

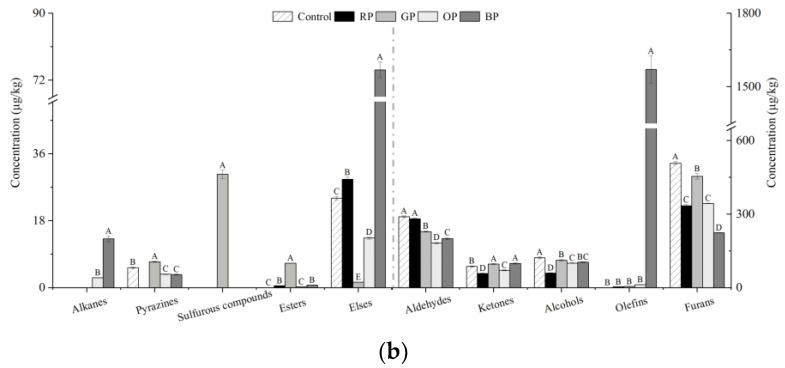
Concentration of volatile compounds. (**a**) Total relative contents of volatile compounds in spices; (**b**) concentrations of volatile compounds in extrudates. OP: Onion powder; RP: Red pepper powder; BP: Black pepper; GP: Garlic powder; Columns with different letters (A–D) indicate significant difference between samples (α = 0.05 level).

Subsequently, the volatile compounds of spices and extrudates were analyzed using HS-SPME-GC-MS (Table S1). The volatile compounds in spices were divided into 10 categories based on their functional groups (Figure 4a). The results of the types of volatile flavor compounds showed that the total number of compounds noted in red pepper, garlic, onion, and black pepper powders were 70, 35, 39, and 23, respectively, including aldehydes (n = 6, 0, 6, and 0), ketones (n = 12, 3, 1, and 2), alcohols (n = 13, 4, 3, and 1), alkanes (n = 8, 7, 11, and 3), pyrazines (n = 4, 0, 0, and 0), sulfurous compounds (n = 1, 8, 13, and 0), olefins (n = 4, 6, 2, and 13), furans (n = 1, 0, 0, and 0), esters (n = 4, 2, 1, and 0), and others (n = 17, 5, 2, and 3). The total relative contents of the four spices are shown in Figure 5a. Sulfurous compounds were the most abundant volatile compounds in both onion and garlic powders, accounting for 61.5% and 68.5%, respectively, of the total compounds detected. The most abundant volatile compounds in black pepper were olefins, and their total relative content was 82.9%, which was consistent with the results of a previous study that high olefin concentrations were noted in black pepper with and without skin [39]. The additions of spices are welcomed owing to their richness in pleasant volatile compounds and their relatively good overall flavor sensed by consumers. Spices can mask some off-flavors, as evaluated in Section 3.1. However, the original volatile compounds may be changed or even not detectable following extrusion; therefore, their potential influences on the volatile compounds of final extrudates should be further investigated.

As shown in Figure 5b, different kinds and concentrations of volatile compounds are noted in spice-treated extrudates owing to the differences of compounds in raw spice powder. A total of 41, 36, 48, 35, and 47 compounds were noted in the control and extrudates treated with red pepper, garlic, onion, and black pepper. Aldehydes, ketones, alcohols, and furans were the most abundant volatile compounds in all the samples. 

Compared with the control, the total concentrations of aldehydes in spice-treated samples decreased 5% to 38% more than the control. Aldehydes were reported to originate from the oxidation of unsaturated lipids such as oleic and linoleic acids during oat extrusion [14]. The concentrations of hexanal, a compound defined as a significant contributor to the beany flavor of foods, decreased from 122.54 µg/kg in the control sample to 100.27 and 99.01 µg/kg in garlic and black pepper-treated extrudates, respectively. 

Alcohols were mainly derived from fat oxidation, and the concentrations of total alcohols in extrudates with red pepper, garlic, onion, and black pepper were 60.94, 108.28, 101.83, and 102.58 µg/kg, respectively. They were all lower than the 119.37 µg/kg in the control sample. Among all the alcohols, 1-octen-3-ol was noted in all samples, and it was produced by a lipase-catalyzed reaction and the oxidative decomposition of unsaturated polyunsaturated fatty acids [46]. Additionally, 1-octen-3-ol was one of the volatile compounds that would lead to off-flavor in plant-based foods, and it had a low threshold so that its decrease would lead to better flavor profiles of extrudates. In control extrudates and red-pepper-, garlic-, onion-, and black-pepper-treated extrudates, the concentrations of 1-octen-3-ol were 31.81, 25.21, 33.34, 31.06, and 15.41 µg/kg, respectively. 

Ketones were generated from the thermo-oxidative degradation or the Maillard reaction of unsaturated fatty acids. The odor thresholds of ketones were generally high; therefore, their influences on extrudates were less pronounced. The total concentrations of ketones in extrudates decreased by 22.3% and 33.3% in red-pepper- and onion-treated extrudates. The result showed that 2-heptanone, 2-octanone, and 2-decanone changed the most in all ketones. Thus, their change may significantly affect extrudates’ flavor. Liu et al. [47] demonstrated that 2-heptanone was generated from linoleic acids and would impart blue-cheese-flavored products. 

Only three furans were detected in all samples. Several pathways are involved in the generation of furan including the Maillard reaction, carbohydrate decomposition, and oxidation reactions [42]. Additionally, 2-pentlyfuran accounted for large concentrations in all samples, and its concentrations decreased from 492.22 µg/kg in control to 436.23, 311.99, 340.73, and 223.05 µg/kg in red-pepper-, garlic-, onion-, and black-pepper-treated extrudates. 

Olefins and alkanes were reported to be representative of fruity, spice, citrus, and sweet flavors. They were the main volatile compounds noted in black pepper, and their concentrations were obviously enhanced in black-pepper-treated extrudates [48].

Sulfurous compounds were only noted in extrudates with garlic powder. Diallyl disulfide, with a meaty and salty flavor, was observed, and would benefit meaty flavor in meat analogs owing to its low odor threshold [49]. 

Ester compounds, which provide samples with fruity flavor, have low odor threshold values, and they can be synthesized by the esterification reaction. The pyrazine concentrations in extrudates decreased from 33% to 73.2% with the addition of spices. Pyrazines were mainly derived from the Strecker degradation of the Maillard reaction [50], and they had unique sensory properties described as baked cereal, roast, and meat odors so that they could significantly affect the sensory characteristics of vegetable protein-based foods [51]. 

Overall, the concentrations of volatile compounds of extrudates, which were reported to be related to further flavor deterioration including oxidation reactions, the Maillard reaction, and carbohydrate decomposition were decreased by the addition of spices. Moreover, the inhibitory effects were the most obvious in black- and red-pepper-treated samples, and those decreases were consistent with the higher antioxidative abilities of black and red pepper, as evaluated in Section 3.2. It could be summarized that spices could be used in extrusion to reduce off-flavor volatile compounds by retarding further flavor deterioration reactions that lead to the generation of aldehydes, alcohols, and the Maillard reactions, which lead to furan generation; most of them would have negative impacts on the total flavor profiles of extrudates. Additionally, to improve the total flavor of extrudates, spices could directly impart their inherent good flavors such as alkanes and olefins to extrudates. However, the pyrazine concentrations with a meaty, roasted flavor and esters with a fruity flavor also decreased; therefore, spices could bring a few undesired effects on flavor as well.

#### 3.3.2. Correlation Analysis

**Figure 6:**
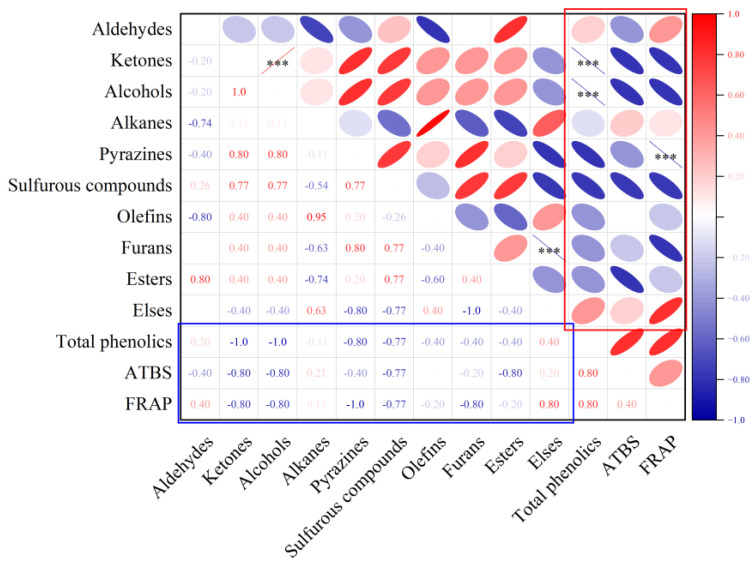
Correlation between volatile compounds and antioxidative abilities of spices. (*** = 0.001 level).

The relationships between different groups of flavor compounds in extrudates and the antioxidative abilities of spices are shown in Figure 6. Furthermore, the antioxidative capacity was one of the main functional properties displayed in phenolic compounds, and the content of total phenolics in different spices was also negatively correlated with the ketones and alcohols (*p* < 0.001). Aldehydes, ketones, and furans were reported to be generated from reactions including oxidation of free fatty acids (e.g., linoleic and linolenic acids) or the Maillard reactions, and most of them were defined as off-flavor volatile compounds in soy-based products [13,50]. It can be speculated that the addition of spices in the extrusion process can inhibit partial oxidative reactions, which would lead to the generation of off-flavor compounds in extrudates, and spices with higher concentrations of phenolic compounds can better retard flavor deteriorations during extrusion. What is more, the concentration of pyrazines negatively correlated with the FRAP value of spices. In conclusion, the total concentrations of some volatile compounds were significantly correlated with the antioxidative abilities and the total phenolics of spices.

#### 3.3.3. PCA

**Figure 7:**
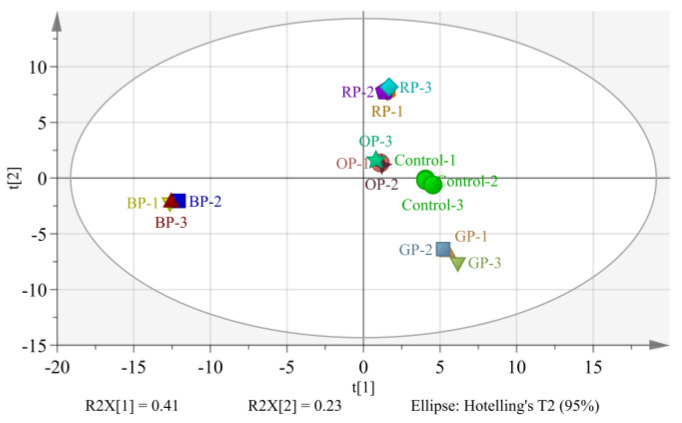
Score plot of PCA of volatile compounds of control extrusion products and those with spices.

The score and load plot of extrudates added with different spices is shown in Figure 7. It can be observed from the figure that the contribution rate of the total variance of the first principal component is 41%, and that of the second principal component is 23%, which is 64% in total, indicating that the off-flavor contents were suitable for PCA, and the analysis method was reliable and effective. The extrudates with different spices were distributed in different quadrants, among which the extrudates with red pepper and onion powders were at the position between the first and second quadrants. Garlic- and black-pepper-powder-treated extrudates were located in the third and fourth quadrants, respectively, and the sample points of black-pepper-treated extrudates were located at the junction of the first and fourth quadrants, whereas the control samples were located at the junction of the first and fourth quadrants. Obvious separation tendencies were noted between black-pepper-, garlic-, and onion-treated samples, and an obvious separation trend in PC1 direction between the sample with onion powder and the control was observed, indicating that adding different spices had significant effects on both contents and compositions of volatile compounds in extrudates.

#### 3.3.4. OAV Analysis of the Sensory Characteristics of Volatile Compounds

OAV stands for odor activity value, and it is calculated from the concentration of the compound and its threshold value in a certain medium. OAV value was used to describe the intensity and effects of compounds on the total flavor of samples. The volatile flavor compounds in the samples of extrudates were analyzed using the OAV method, and the contributions of each volatile compound to extrudates were evaluated (Table 2). A total of 109 volatile compounds were detected in the extrudates samples. There were 29 flavor compounds with OAVs of ≥1, including aldehydes (8), alcohols (7), olefins (5), furans (3), ketones (2), sulfurous compounds (2), and indole and a phenol substance. Substances including hexanal, octanal, decanal, (E, E)-2,4-decadienal, 2-pentlyfuran, 1-octen-3-ol, 1-hexanol, 1-octanol, 1-caryophyllene, (+)-4-carene, and pinene were with OAVs of >10; therefore, they could be considered the main aroma-active compounds in each spice-treated extrudate sample. Among them, 1-caryophyllene, 1-octen-3-ol, 2-pentlyfuran, hexanal, and nonanal were the main aroma-active compounds in all samples.

Aldehydes can largely influence the total flavor of samples owing to their relatively low threshold. The extrusion of aldehydes such as hexanal, octanal, nonanal, 2-octenal, and (E, E)-2,4-Decadienal, may be generated from the oxidation of substances, including free fatty acids, linoleate, linoleic acid, and n-6 polyunsaturated fatty acids [14,51]. In this study, the OAVs of some of them decreased with the addition of spices owing to their antioxidative abilities, as stated in Section 3.2. Those substances were reported as grassy, rubbery, fatty, and oily, and they were unexpected in the sample; therefore, spices may have positive influences on the total volatile flavor of extrudates by partially inhibiting their generation during extrusion. However, the antioxidative abilities of spices may also have a little adverse impact on extrudates’ flavor. The OAV of a compound, a pleasant chicken-like flavor compound (E, E)-2,4-decadienal, also decreased since it was an oxidation product of linoleic and linolenic acids, and the reaction was inhibited by the addition of spices [52,53].

Moreover, alcohols such as 1-octen-3-ol, 1-hexanol, 1-heptanol, and 1-nonanol were reported to be derived from β-ketoacid decarboxylation or the β-oxidation of fatty acids [54]. Among them, 1-octen-3-ol was reported to be one of the off-flavors that lead to beany flavor, which would damage the sensory characteristics in soy-based foods, such as soy milk and textured sausages analog [35,37]. Furthermore, two exogenous alcohols including terpineol and linalool were observed in onion- and black-pepper-treated extrudates and were not observed in control samples. Both of them may have positive influences on the flavor of extrudates. First, terpineol was reported to be the flavor of clove and pine, whereas linalool was reported as flowery; therefore, both may bring pleasant flavors to extrudates. Second, most of the detected aldehydes with unpleasant flavor descriptions in this study may deteriorate the flavor of extrudates. It was reported that the ranking of the tendency of flavors in different functional groups to combine with protein was alcohols > aldehydes, indicating that alcohols may combine easier with protein than aldehydes, and aldehydes will be released because their binding sites were taken by alcohols. Additionally, the aldehyde concentrations may be reduced owing to the volatilization that occurred at the exit of cooling zone; therefore, more volatilization of aldehydes may have a positive impact on the overall flavor [3,55].

The five olefins were defined as aroma-active compounds in extrudates with added black pepper, and they may benefit the total sensory characteristics of extrudates since most of them were described as spicy, citrus, lemon, and sweet flavors, respectively. Substances including 3-carene (5.57%), (+)-4-carene (0.88%), d-limonene (4.35%), and caryophyllene (14%) detected extrudates derived from the added black pepper powder. They were detected in the black pepper powder that was used in this study, and they were previously reported to be noted in certain amounts in black pepper by several studies [39,48].

Among all the furans, 2-pentlyfuran, described as grassy and beany, was detected in all samples, and it can be generated from the decomposition of carbohydrates, the Maillard reaction, thermal degradation of amino acids, and oxidative pyrolysis of linoleic acid and 2-decadienal [42,56]. The OAVs of 2-pentlyfuran decreased by approximately 22–55% in the spice-treated samples because the phenolics with antioxidative abilities in spices suppressed oxidative reactions in extrusion.

The sulfur compounds have very low odor thresholds. They generally have meat, onion, garlic, and sulfide flavors, which is the basic component of various meat flavors. Diallyl disulfide, which accounted for 23.35% of the total volatile compounds in garlic powder, was noted in garlic-powder-treated extrudates. Diallyl disulfide was described as garlic, meaty, or salty; therefore, the flavor of extrudates may be enriched and consumers’ acceptance of meat analogs may be enhanced [57,58].

Additionally, 2,4-Di-tert-butylphenol was reported to be a significant volatile compound contributing to the flavor of soy sauce, and it would bring phenol or fruity flavor to foods [59,60]. The specific contribution of each volatile compound to the overall flavor profile was understood by combining the identified volatile flavor compounds with their flavor thresholds.

As shown in Table 2, compounds with >1 OAV value would significantly influence the total flavor of extrudates, so we summed up the OAV value of compounds with >1 OAV value. The total OAV value of off-flavor volatile compounds in the control, onion, red-pepper, black-pepper-, and garlic-treated extrudates were 286, 258.16, 204.44, 104.17, and 218.76, respectively. Among them, there were maximum changes in nonanal, 2-pentlyfuran, and many other compounds, which may be derived from lipid oxidation. The total OAV value of pleasant-flavor volatile compounds in the control-, onion-, red-pepper-, black-pepper-, and garlic-treated extrudates were 30.3, 134.83, 3.29, 18577.99, and 557.01, respectively. Only extrudates treated with onion had lower total OAV values than that of the control. The types and contents of pleasant-flavor volatile compounds changed significantly in the other three samples treated with red pepper, black pepper, and garlic. In conclusion, the flavor profiles of extrudates were both altered by the masking effects of characteristic flavors in spices and the decrease in the oxidative-related flavor generation by the antioxidative abilities of spices.

## 4. Conclusions

Herein, the total concentration of off-flavor volatile compounds varied in commercial meat analogs produced by different processing. Based on the analyses of 50 commercial meat analog products, it can be inferred that the addition of spices such as black pepper, red pepper, onion, and garlic enhanced both their sensory characteristics and consumers’ preferences. Adding black pepper, red pepper, garlic, and onion during extrusion reduced the generation of some volatile compounds associated with heat-related treatments. The addition of these spices during extrusion enriched the aroma-active compounds of extrudates by providing them with pleasant flavors including alkanes and olefins which were commonly defined as pleasant flavors. In conclusion, adding spices can partially retard the generation of some off-flavor volatile compounds such as aldehydes, alcohols, and ketones, and it can also enrich the flavor profiles of extrudates. The result of this study can be utilized to produce extrudates with better sensory quality, which can be used in the production of commercial meat analog products. Further research on the synergetic effect of spices and antioxidants on the off-flavor generation and flavor profile improvement during extrusion is warranted.

## Figures and Tables

**Table 1:** Off-flavor volatile compounds in commercial products.

Retention Time	Compounds	CAS	Odor Description
3.531	Ethanol	64-17-5	alcohol
6.032	Hexanal	66-25-1	grassy, fishy
8.058	Heptanal	111-71-7	fatty, oily
9.067	2-Pentylfuran	3777-69-3	fragrant, waxy, mushroom
9.313	1-Pentanol	71-41-0	spicy
9.956	Octanal	124-13-0	fatty, spicy
9.987	2-Octanone	111-13-7	soapy, fruity
10.598	2-Heptenal	2463-63-0	fatty
11.165	1-Hexanol	111-27-3	salty, beany, potato
11.821	2-Nonanone	821-55-6	fatty
11.86	Nonanal	124-9-6	waxy, fatty
12.114	3-Octen-2-one	1669-44-9	earthy, pungency
12.437	2-Octenal	2363-89-5	vegetable, cucumber, fatty
12.691	Acetic acid	64-19-7	acid
12.841	1-Octen-3-ol	3391-86-4	mushroom, grassy
13.628	Decanal	112-31-2	oily
14.181	(E)-2-Nonenal	18829-56-6	cucumber, woody
14.721	3,5-Octadien-2-one	30086-02-3	grassy, green
15.849	Trans-2-decenal	3913-81-3	Rosin, grassy
16.129	1-Nonanol	143-08-8	fatty, citrus
18.216	(E, E)-2,4-Decadienal	25152-84-5	spicy, fatty
18.608	Hexanoic acid	142-62-1	cheesy, pungency
21.368	Octanoic acid	124-07-2	stringency

**Table 2:** Aroma-active compounds in extrudates with and without spices.

	Compounds	OAV				
		Control	OP	RP	BP	GP
Off-flavor compounds in extrudates	Hexanal	24.51	24.56	27.39	19.8	24.28
	Octanal	17.5	17.02	12.78	N.D.	12.44
	Nonanal	26.6	16.26	18.32	15.86	19.89
	Decanal	68.5	85.2	51.9	N.D.	47.2
	2-Octenal	1.75	1.69	1.09	1.12	1.21
	3-Octen-2-one	5.47	N.D.	3.63	2.65	3.81
	Indole	1.77	N.D.	1.67	N.D.	N.D.
	2-Butylfuran	1.7	N.D.	2.73	N.D.	1.93
	2-Pentlyfuran	84.87	58.75	53.79	38.46	65.8
	2-Ethylfuran	N.D.	N.D.	5.93	N.D.	N.D.
	1-Octen-3-ol	31.81	31.06	25.21	15.41	25
	1-Heptanol	2.22	2.56	N.D.	1.12	1.74
	1-Nonanol	1.23	1.33	N.D.	N.D.	N.D.
	1-Hexanol	18.07	19.73	N.D.	9.75	15.46
	**Sum**	**286**	**258.16**	**204.44**	**104.17**	**218.76**
Pleasant-flavor compounds in extrudates	Terpineol	N.D.	5.51	N.D.	N.D.	N.D.
	Linalool	N.D.	N.D.	N.D.	7.39	N.D.
	2-Nonanone	1.83	3.33	1.06	N.D.	1.12
	1-Caryophyllene	14.06	68.59	N.D.	14,997.34	43.13
	3-Carene	N.D.	N.D.	N.D.	3389.09	N.D.
	D-Limonene	N.D.	N.D.	N.D.	8.21	N.D.
	(+)-4- Carene	N.D.	N.D.	N.D.	124.55	N.D.
	Pinene	N.D.	N.D.	N.D.	11.04	N.D.
	2,4-Di-tert-butylphenol	N.D.	N.D.	N.D.	4.1	N.D.
	Diallyl sulfide	N.D.	N.D.	N.D.	N.D.	501.8
	Diallyl disulfide	N.D.	N.D.	N.D.	N.D.	1.02
	Benzaldehyde	1.54	1.01	N.D.	N.D.	1.14
	Pentanal	N.D.	N.D.	2.23	N.D.	N.D.
	(E, E)-2,4-Decadienal	12.87	10.57	N.D.	N.D.	8.8
	1-Octanol	N.D.	45.82	N.D.	36.27	N.D.
	**Sum**	**30.3**	**134.83**	**3.29**	**18,577.99**	**557.01**

OP: Onion powder; RP: Red pepper powder; BP: Black pepper; GP: Garlic powder; N.D. means not detected. Sum means the total OAV value of off-flavor and pleasant-flavor volatile compounds in extrudates.

## Data Availability

The data presented in this study are available on request from the corresponding author.

## Supplementary Materials

The following supporting information can be downloaded at: https://www.mdpi.com/article/10.3390/foods12081650/s1, Table S1: Flavor compounds in spices and extrudates.
