# Supplementary material for: Influences of Spices on the Flavor of Meat Analogs and Their Potential Pathways
Source: Foods. 2023 Apr 15;12(8):1650. doi: 10.3390/foods12081650 (PMC10137762; doi:10.3390/foods12081650)
Supplement: Supplementary file 1 [file foods-12-01650-s001.zip › foods-2249287-supplementary.pdf]

Table S1. Flavor compounds in spices and extrudates.

	Relative contents (%)				Concentrations (μg/kg)				
	RP	GP	OP	BP	Control	GP	OP	RP	BP
**Aldehydes**									
Pentanal							5.9±0.64^{b}	20.03±1.02^{a}	
Octanal					10.27±0.13^{a}	8.02±0.19^{b}	9.99±0.44^{a}	7.5±0.68^{b}	
Hexanal	0.24		0.07		122.54±2.67^{b}	100.27±4.34^{c}	122.79±3.39^{b}	136.93±7.31^{a}	99.01±3.16^{c}
Nonanal	0.75		0.27		29.26±0.66^{ab}	26.91±0.52^{b}	16.89±2.27^{a}	20.15±0.85^{c}	17.45±0.81^{d}
Decanal					6.85±0.23^{b}	6.55±0.5^{b}	8.52±0.56^{a}	5.19±0.23^{c}	
2-Furancarboxaldehyde	1.32		1.41		29.46±1.78^{a}	22.36±0.65^{b}	22.47±1.65^{b}	23.16±1.67^{b}	12.62±0.39^{c}
Dodecanal							5.27±2.22^{a}		
Tridecanal					3.06±0.24^{a}	2.4±0.28^{b}		2.44±0.18^{b}	
Benzaldehyde					64.07±2.06^{a}	41.91±4.12^{b}		41.04±0.95^{b}	61.32±2.75a
1,3-Cyclohexadiene-1-carboxaldehyde, 2,6,6-trimethyl-	2.46								
Benzaldehyde, 2,4-dimethyl-	1.95								
5-Methyl-2-phenyl-2-hexenal	0.07								
2-Butenal, 2-methyl-			1.43						
2-Pentenal, 2-methyl-			0.84				3.2±0.12^{a}		
Benzaldehyde, 4-methoxy-			0.42						
Piperonal				1.45					
2-Ethyl-2-hexenal					26.96±0.63^{a}	22.45±1.73^{b}		18.32±0.8^{c}	2.82±0.04^{d}
2-Octenal					5.24±0.19^{a}		5.08±0.22^{a}	3.28±0.34^{b}	3.35±0.15^{b}
2-Octenal, 2-butyl-					2.46±1.49^{ab}	2.24±1.76^{ab}	2.88±1.08^{a}	4.45±0.34^{a}	
(E, E)-2,4-Decadienal					0.9±0.06^{a}		0.74±0.04^{b}		
H-Pyrrole-2-carboxaldehyde					0.57±0.03^{b}			0.87±0.11^{a}	
**Number (total)**	6	0	6		12	9	11	11	7
**Ketones**									
2-Tridecanone		1.24							
2-Pentadecanone						0.19±0.01^{a}			
2-Undecanone			3.15			15.25±4.73^{a}			
2-Tridecanone			0.85						
2-Heptanone					53.67±1.31^{a}	35.95±1.95^{b}	25.01±2.98^{c}	38.41±1.3^{b}	27.33±0.81^{c}
2-Octanone					12.23±0.22^{a}	9.27±0.42^{b}	10.48±0.89^{a}	6.8±0.25^{c}	
2-Decanone					5.91±0.32^{a}	5.55±0.41^{ab}	5.17±0.33^{b}	3.58±0.08^{c}	2.85±0.08^{d}
2-Nonanone					9.16±0.42^{b}	9.78±0.22^{b}	16.65±1.1^{a}	5.28±0.25^{c}	
Acetoin	0.33								
2,3-octanedione						5.02±0.46^{a}			
3-Octen-2-one					8.21±0.4^{a}	7.44±0.24^{b}		5.45±0.06^{c}	3.97±0.1^{d}
Trans-3-Nonen-2-one					9.11±0.21^{a}	8.61±0.94^{a}	8.48±0.12^{a}		6.74±0.22^{b}
(E)-3-Penten-2-one	0.45						3.51±0.69^{a}		
5-Hepten-2-one, 6-methyl-	3.08								
2-Nonen-4-one	0.32								
Ethanone, 1-(2-furanyl)-	3.12								
2-Cyclopenten-1-one, 3,4,4-trimethyl-	0.17								
6-Methyl-3,5-heptadiene-2-one	5.16								
2,6,6-Trimethyl-2-cyclohexene-1,4-dione	0.55								
(E)-5,9-Undecadien-2-one, 6,10-dimethyl-	4.08								
3-Buten-2-one, 4-(2,6,6-trimethyl-1-cyclohexen-1-yl)-	2.16								
2(3H)-Furanone, dihydro-5-pentyl-	0.15								
4H-Pyran-4-one, 2,3-dihydro-3,5-dihydroxy-6-methyl-	0.70								
5,9-Undecadien-2-one, 6,10-dimethyl-, (E)-		0.26							

	Relative contents (%)				Concentrations (μg/kg)				
	**RP**	**GP**	**OP**	**BP**	**Control**	**GP**	**OP**	**RP**	**BP**
Ethanone, 1-(1H-pyrrol-2-yl)-		0.21				0.81±0.15^{a}			
4H-Pyran-4-one, 2,3-dihydro-3,5-dihydroxy-6-methyl-			0.25						
(4R,5R)-4-hydroxy-5-isopropyl-2-methylcyclohex-2-enone2-enone				0.47					
2-Cyclohexen-1-one, 4-hydroxy-3-methyl-6-(1-methylethyl)-, trans-				0.09					
2,6-Di(tert-butyl)-4-hydroxy-4-methyl-2,5-cyclohexadien-1-one						0.38±0.03^{b}		1.06±0.19^{a}	
2,4-Cycloheptadien-1-one, 2,6,6-trimethyl-									53.39±38.3^{a}
4,7,7-Trimethylbicyclo [4.1.0] hept-3-en-2-one									2.4±0.16^{a}
**Number (total)**	12	3	1	2	6	11	6	6	6
**Alcohols**									
1-Hexanol	0.16				36.13±0.28^{b}	28.82±0.4^{c}	39.46±0.74^{a}		19.49±0.77^{e}
3-Hexanol					3.76±0.19^{a}	2.3±1.29^{b}			
2,3-Butanediol	7.95								
2-Furanmethanol	3.40		0.49		6.62±0.53^{a}	5.15±0.24^{b}		6.29±0.67^{a}	
3,5-Octadien-2-ol	0.08								
2-Hexadecanol	0.04								
Geraniol	0.16								
Benzyl alcohol	0.37	0.24	0.40						
Phenylethyl Alcohol	0.52		0.28			1.34±0.39^{a}			
2-Propen-1-ol		8.00							
1-Nonanol					2.46±0.17^{b}	3.18±0.21^{a}	2.66±0.22^{b}	1.88±0.08^{c}	
1-Pentanol					1.54±0.07^{a}	1.19±0.04^{b}		1.04±0.21^{b}	
1-Dodecanol					0.43±0.05^{a}				
1-Octanol							5.04±0.36^{a}		3.99±0.19^{b}
2-Octanol									0.87±0.05^{a}
1-Heptanol					6.66±0.29^{b}	6.24±0.04^{b}	7.69±0.43^{a}		3.37±0.09^{c}
2-Heptanol					6.09±0.24^{b}	6.97±0.91^{b}		12.09±0.54^{a}	2.67±0.19^{c}
Santalol									2.15±0.06^{a}
Linalool	0.65	0.58					2.27±0.3^{b}	0.45±0.03^{b}	44.34±2.36^{a}
1-Octen-3-ol	1.46				31.81±0.97^{a}	33.34±2.03^{a}	31.06±1.95^{a}	25.21±2.46^{b}	15.41±0.84^{c}
Isospathulenol				0.46					6.27±2.37^{a}
Trans-2-Dodecen-1-ol	1.02								
2-Furanmethanol, 5-methyl-	1.59								
3-Cyclohexen-1-ol, 4-methyl-1-(1-methylethyl)-, (R)-		0.35							
2-Isopropyl-5-methyl-1-heptanol	0.24								
4-Methyl-2-pentanol					5.59±0.18^{a}	4.13±0.51^{b}		5.12±0.25^{a}	
1-Adamantanol					15.95±0.61^{a}	12.49±0.57^{b}	8.05±0.23^{c}	7.13±0.32^{cd}	6.18±0.87^{d}
2-Ethyl-1-hexanol					2.33±0.13^{a}	1.88±0.18^{b}	2.35±0.12^{a}	1.73±0.02^{b}	
(-)-alpha-Terpineol							3.25±3.48^{a}		
p-Mentha-1,5-dien-8-ol									9.19±0.37^{a}
2-Cyclohexen-1-ol, 2-methyl-5-(1-methylethenyl)-, cis-									0.76±0.06^{a}
Caryophylla-4(12),8(13)-dien-5. alpha. -ol									7.38±0.08^{a}
2,4-Dimethylcyclohexanol						1.25±0.01^{a}			
**Number (total)**	13	4	3	1	12	13	9	9	12
**Alkanes**									
Undecane	1.18								
Dodecane	2.72		1.23						
Tetradecane	0.66	0.61							
Pentadecane							2.75±0.1^{a}		
Hexadecane		0.24	1.03						

	Relative contents (%)				Concentrations (μg/kg)				
	RP	GP	OP	BP	Control	GP	OP	RP	BP
Heptadecane	0.34	0.28							
Cyclododecane		0.25							
Dodecane, 4-methyl-	0.05								
Heptadecane, 2,6,10,15-tetramethyl-	0.02								
Dodecane, 2,6,11-trimethyl-	0.24								
3-methyl-1,2-Cyclopentanedione	0.89								
1-Allyl-2-isopropyldisulfane		0.84							
Hexadecane, 2-methyl-		0.17							
Cyclohexane, octyl-		0.36							
Decane, 2,6,8-trimethyl-			0.65						
1-Allyl-2-isopropyldisulfane			4.09						
Cyclopentane, undecyl-			0.17						
Cyclopropane, nonyl-			0.31						
(E)-1-(Prop-1-en-1-yl)-3-propyltrisulfane			13.89						
1-Methyl-2-(1-(propylthio) propyl) disulfane			0.78						
6-Ethyl-4,5,7,8-tetrathianonane			0.23						
6-Ethyl-4,5,7,8-tetrathianonane			0.23						
4,7-Diethyl-1,2,3,5,6-pentathiepane			0.10						
Bicyclo[3.1.1]heptane, 6,6-dimethyl-2-methylene-, (1S)-				2.61					
(1S,2R,4R,7R)-4-Isopropyl-7-methyl-3,8-dioxatricyclo [5.1.0.02,4] octane				1.60					
(1R,3E,7E,11R)-1,5,5,8-Tetramethyl-12-oxabicyclo [9.1.0] dodeca-3,7-diene				1.37					
Cyclohexane, 1-ethenyl-1-methyl-2-(1-methylethenyl)-4-(1-methylethylidene)-									6.68±2.74^{a}
Undecane, 2,4-dimethyl-									1.94±0.12^{a}
Heptadecane, 8-methyl-									5.27±0.43^{a}
**Number (total)**	8	7	11	3	0	0	1	0	3
**Pyrazines**									
Pyrazine, 2,5-dimethyl-					3.68±0.62^{a}	2.99±0.08^{ab}	3.73±0.5^{a}		2.86±0.01^{b}
Pyrazine, 2,6-dimethyl-	0.75								
Pyrazine, 2-ethyl-5-methyl-	0.19								
Pyrazine, 2-ethyl-6-methyl-					1.89±0.13^{b}	3.85±0.53^{a}			0.76±0.03^{c}
Pyrazine, trimethyl-	0.85								
Pyrazine, tetramethyl-	1.56								
**Number (total)**	4	0	0	0	2	2	1	0	2
**Sulfurous compounds**									
Diallyl disulphide		23.38				4.31±0.28^{a}			
Dimethyl trisulfide	0.30	0.98	0.18						
Disulfide, methyl 2-propenyl		4.26							
Trisulfide, methyl 2-propenyl		3.76							
Trisulfide, di-2-propenyl		13.89				25.09±0.73^{a}			
2-Vinyl-4H-1,3-dithiine		2.62							
Disulfide, methyl propyl			1.57						
(E)-1-Methyl-2-(prop-1-en-1-yl)disulfane			1.61						
Dipropyl disulfide			13.46						
Dipropyl Trisulfide			24.51						
Trisulfide, methyl propyl			11.11						
Trisulfide, methyl 2-propenyl			0.39						
(E)-1-(Prop-1-en-1-yl)-2-propyldisulfane			3.41						
(E)-1-Allyl-2-(prop-1-en-1-yl) disulfane			0.46						
Disulfide, methyl 1-(methylthio)propyl			1.28						
1-(1-(Methylthio) propyl)-2-propyldisulfane			1.41						
Disulfide, methyl 2-propenyl			0.23						
Disulfide, methyl 1-(1-propenylthio) propyl			1.93						

	Relative contents (%)				Concentrations (μg/kg)				
	**RP**	**GP**	**OP**	**BP**	**Control**	**GP**	**OP**	**RP**	**BP**
3H-1,2-Dithiole		18.62							
4-Methyl-1,2,3-trithiolane		1.03							
**Number (total)**	1	8	13	0	0	2	0	0	0
**Olefins**									
Myrcene	0.12								
D-Limonene	0.26		0.06	4.35			8.22±0.17^{b}		279.25±19.62^{a}
Caryophyllene	0.33	0.32		14					
Propene		4.01							
Copaene		0.36							
3-Carene				5.57					149.12±14.84^{a}
Beta-Pinene				2.42					
Gamma-Terpinene				0.19					
(+)-4-Carene				0.88					5.48±0.16^{a}
Humulene				8.41					
Aromandendrene				6.61					
Caryophyllene oxide				2.47					7.69±8.24^{a}
α-Caryophyllene							4.39±0.12^{a}		
3-Ethyl-2-methyl-1,3-hexadien								3.41±0.33^{a}	
Copaene				18.88					66.26±2.86^{a}
cis-(-)-1,2-Epoxy-p-menth-8-ene				0.04					
Alpha-Cubebene				0.18					
4-tert-Butoxystyrene						5.07±1.65^{a}			
1,3-Cyclohexadiene, 1-methyl-4-(1-methylethyl)-	0.26								
3-Vinyl-1,2-dithiacyclohex-4-ene		9.42							
6-Methyl-4,5,8-trithia-1,10-undecadiene		0.20							
Bicyclo[3.1.0]hexan-3-ol, 4-methylene-1-(1-methylethyl)-, (1.alpha.,3.alpha.,5.alpha.)-									6.96±0.32^{a}
Cyclohexene, 4-ethenyl-4-methyl-3-(1-methylethenyl)-1-(1-methylethyl)-, (3R-trans)-			0.15	2.00					
Bicyclo[7.2.0]undec-4-ene, 4,11,11-trimethyl-8-methylene-									28.79±0.84^{a}
(1S,2E,6E,10R)-3,7,11,11-Tetramethylbicyclo [8.1.0] undeca-2,6-diene									2.17±0.16^{a}
4-isopropyl-1,6-dimethyl-1,2,3,4-tetrahydronaphthalene									10.39±0.44^{a}
**Number (total)**	4	6	2	13	1	1	1	1	9
**Furans**									
2-Ethylfuran								13.63±0.14^{a}	
2-Pentylfuran	0.95				492.22±6.34^{a}	436.23±8.81^{b}	340.73±13.13^{c}	311.99±9.7^{d}	223.05±1.59^{e}
2-Butylfuran					8.52±0.11^{b}	7.38±2.22^{b}	4.37±0.17^{c}	13.66±0.71^{a}	
**Number (total)**	1	0	0	0	2	2	2	3	11
**Esters**									
Acetic acid, 2-ethylbutyl ester	0.02								
Hexadecanoic acid, methyl ester	0.15								
Dodecanoic acid, methyl ester		0.80							
Hexadecanoic acid, methyl ester		0.25	0.12			0.46±0.01^{a}			
Benzoic acid, ethyl ester					3.6±0.18^{a}	3.77±0.31^{a}			
Isopropyl palmitate					0.56±0.09^{b}	1±0.15^{a}			
Isopropyl myristate					0.56±0.11^{a}		0.24±0.02^{b}	0.51±0.18^{a}	0.67±0.05^{a}
Estergel						1.28±0.08^{a}			
Benzene, 1-methyl-3-(1-methylethyl)-	0.11								
Butanoic acid, 2,2-dimethyl-	0.03								
**Number (total)**	4	2	1	0	3	4	1	1	1
**Else**									

	Relative contents (%)				Concentrations (μg/kg)				
	**RP**	**GP**	**OP**	**BP**	**Control**	**GP**	**OP**	**RP**	**BP**
2-Propylpyridine						0.54±0.31^{a}			
Pyridine, 2,3-dimethyl-			0.26						
1H-Pyrrole, 1-(2-furanylmethyl)-	0.07								
Ethanone, 1-(1H-pyrrol-2-yl)-	8.29								
Maltol	1.01								
Phenol, 2-methoxy-		0.79							
Eugenol		0.61							
Butylated Hydroxytoluene					21.29±1.13^{a}		10.03±0.3^{d}	18.15±1.38^{b}	16.11±0.42^{c}
2-Methoxy-4-vinylphenol					0.68±0.09^{b}	0.46±0.03^{c}	0.3±0.03^{d}	0.84±0.08^{a}	
2,4-Di-tert-butylphenol					1.1±0.2^{a}	0.59±0.04^{c}		0.66±0.01^{bc}	0.82±0.04^{b}
Indole					0.53±0.08^{a}	0.4±0.01^{b}		0.5±0.05^{a}	
o-Cymene				1.48					74.84±3.11^{a}
p-(1-Propenyl)-toluene				0.39					
Naphthalene, 1,2,4a,5,8,8a-hexahydro-4,7-dimethyl-1-(1-methylethyl)-, [1S-(1. alpha. 4a. beta,8a. alpha.)]-				4.88					46.59±1.56^{a}
Naphthalene, 1,2,3,4,4a,7-hexahydro-1,6-dimethyl-4-(1-methylethyl)-									2.05±0.2^{a}
Octanoic acid	0.26								
Nonanoic acid	0.06								
Heptanoic acid	0.13								
Acetic acid	22.20	0.18						9.07±0.69^{a}	
Formic acid	0.93								
Propanoic acid	0.40								
Hexanoic acid	6.32								
Dodecanoic acid	0.03								
Pentanoic acid, 4-methyl-	0.19								
Divinyl sulfide	0.05								
Succinimide	0.04								
Ethoxyquin	0.06								
Thiophene, 2,4-dimethyl-			2.17						
**Number (total)**	17	5	2	3	4	4	2	4	5
